# Small Sample Hyperspectral Image Classification Based on the Random Patches Network and Recursive Filtering

**DOI:** 10.3390/s23052499

**Published:** 2023-02-23

**Authors:** Denis Uchaev, Dmitry Uchaev

**Affiliations:** 1 Laboratory of Intelligent Systems for Processing Spatial Data, Moscow State University of Geodesy and Cartography (MIIGAiK), Moscow 105064, Russia; 2 Department of Space Monitoring and Ecology, Moscow State University of Geodesy and Cartography (MIIGAiK), Moscow 105064, Russia

**Keywords:** hyperspectral data, few-shot learning, deep features, convolution kernels, edge-preserving filtering

## Abstract

In recent years, different deep learning frameworks were introduced for hyperspectral image (HSI) classification. However, the proposed network models have a higher model complexity, and do not provide high classification accuracy if few-shot learning is used. This paper presents an HSI classification method that combines random patches network (RPNet) and recursive filtering (RF) to obtain informative deep features. The proposed method first convolves image bands with random patches to extract multi-level deep RPNet features. Thereafter, the RPNet feature set is subjected to dimension reduction through principal component analysis (PCA), and the extracted components are filtered using the RF procedure. Finally, the HSI spectral features and the obtained RPNet–RF features are combined to classify the HSI using a support vector machine (SVM) classifier. In order to test the performance of the proposed RPNet–RF method, some experiments were performed on three widely known datasets using a few training samples for each class, and classification results were compared with those obtained by other advanced HSI classification methods adopted for small training samples. The comparison showed that the RPNet–RF classification is characterized by higher values of such evaluation metrics as overall accuracy and Kappa coefficient.

## 1. Introduction

Currently, HSI classification is used to solve many Earth remote sensing problems, such as identifying tree species [1,2], estimating crop yields [3,4], and oil spill detection [5,6]. Good HSI classification results are obtained if there is sufficient labeled data for training. However, gathering sufficient labeled training data is often too expensive in terms of economic costs and time. For this reason, in many practical applications, small training samples have to be used for HSI classification. The imbalance between the large number of HSI spectral bands and the limited availability of training samples can be the cause of the Hughes phenomenon, which leads to the risk of overfitting the training data. In this regard, studies aimed to obtain good HSI classification results on small training samples is attracting more and more attention.

The key steps of HSI classification are feature extraction and representation [7,8]. Before the widespread application of deep learning methods, HSI classification is based on the use of hand-crafted (shallow) features, such as local binary patterns [9,10], morphological features [11,12], and fractal-based features [13,14]. However, shallow feature extraction techniques often require careful engineering and domain knowledge of experts, which limits their applications. In contrast, deep learning techniques aim at automatically extracting high-level features from raw data in a hierarchical manner. These features are more discriminative, abstract, and robust than shallow features [8].

In recent years, deep learning methods based on convolutional neural networks (CNNs) have been increasingly applied to HSI classification, because CNNs can effectively capture features from HSI pixels by exploiting the shape, layout, and texture of ground objects, which combines the spatial and spectral information. All CNN-based HSI classification methods can be divided into the following three types: spectral CNN, spatial CNN, and spectral-spatial CNN. For small sample HSI classification, spectral-spatial CNN-based methods are generally applied. These methods make it possible to explore the spectral and spatial HSI information in a unified framework using 2D-CNN [15], 3D-CNN [16,17,18], or some combinations of 1D-CNN, 2D-CNN, and 3D-CNN [19,20,21]. Deep CNN-based models increase the accuracy of HSI classification. However, training becomes harder as network depth increases. Moreover, deeper CNNs easily lead to overfitting with limited training samples. In this regard, in some papers [22,23,24], texture features extracted from the HSI or principal components (PCs) of the HSI are applied as input to the CNN to improve the performance of the network. 

Instead of CNNs, generative adversarial networks (GANs) can be used to classify HSIs under the condition of limited training samples. GAN is built by combining a generator and a discriminator. The generator attempts to generate samples that approximate real samples, and the discriminator attempts to distinguish whether the inputs are generated or real samples. Thus, in [25], a 3D-GAN method based on the use of both spatial and spectral HSI information is presented. [26,27,28] propose semi-supervised GAN-based methods for HSI classification. Some GAN-based methods combine GAN with traditional techniques, such as 3D bilateral filters [26] and conditional random fields [29]. An effective GAN-based method for small sample HSI classification is proposed in [30], which presents a symmetric convolutional GAN based on collaborative learning and attention mechanism (CA-GAN). In CA-GAN, a generator using both collaborative and competitive learning generates high-quality samples. Moreover, in CA-GAN, the discriminator captures global spectral dependencies instead of local correlation captured by convolutional kernels in existing GANs. As shown in [30], CA-GAN outperforms other advanced GAN-based HSI classification methods when the number of training samples is limited. The drawback of GAN-based methods is that GANs are hard to train because the generator and discriminator models are trained simultaneously in a game.

Rich opportunities for small sample HSI classification open up semi-supervised and active learning methods that can use unlabeled training samples in addition to a limited set of labeled samples. These methods are based on the assumption that there is no severe shift between the two data distributions, which are the target domain data and the source domain data. Thus, in [31,32,33,34], graph-based semi-supervised and active learning methods are presented and analyzed, which are characterized by high classification accuracy with limited training samples. In [35], Hu et al. propose a 3D VS-CNN method that uses active learning to construct valuable training samples, which improves the small sample classification. However, semi-supervised and active learning methods do not take into account the domain shift problem (due to different environmental conditions, such as light or atmosphere, the target and source domains usually have a significant spectral shift). 

To overcome the domain shift problem, deep cross-domain methods can be used, which are based on transfer learning and apply characteristics of the source domain to the target domain. Thus, Ref [36] proposes a multitask deep learning method that simultaneously conducts classification and reconstruction in an open world (called MDL4OW) where unknown classes may exist. Ref [37] introduces deep cross-domain few-shot learning (DCFSL) method that combines few-shot learning and a domain adaptation strategy in a conditional adversarial manner together to address the issue that there may be different data distributions between target and source domains. In addition, few-shot learning is executed in source and target classes at the same time, which can not only discover transferable knowledge in the source classes, but also learn a discriminative embedding model to the target classes. However, an open problem of all cross-domain methods is the fitness of external datasets.

In order to solve the few-shot learning problem without involving external datasets, metric learning can be used [38,39,40]. Metric learning allows exhibiting a relationship between two samples through mapping these samples into a metric space. In the metric space, the distance between the samples of same classes will be as close as possible, and the distance between the samples without the same classes is as large as possible. As shown in [39], when the number of training samples is 50 per class, good HSI classification results are obtained using a similarity-based deep metric model (S-DMM) that provides a classification accuracy very close to 100% on test datasets. A disadvantage of metric learning methods is that they are usually very time-consuming. 

In the last few years, Transformer-based methods have also been used to classify HSIs with limited training samples [41,42,43,44,45]. A Transformer is a recently proposed deep-learning model that adopts a self-attention mechanism that weights the significance of each part of input data differently. Unlike CNNs, which are suitable for extracting local features, Transformers are better suited to capture global (long-range) contextual relationships. If very small training samples are used for each class, excellent HSI classification results are obtained by the Transformer-based method from [42]. This method does not use external datasets and combines a GAN, Transformer encoder (TE), and Convolution block in a unified framework that can be denoted as TC-GAN. The proposed method has both a global receptive field provided by the TE and a local receptive field provided by the convolution block. The drawbacks of the Transformer-based methods are that Transformers are generally less capable for extraction of fine-grained local feature patterns [46], and do not fully utilize spatial information [45].

In this paper, a deep learning method is proposed for small sample HSI classification, which is based on the use of RPNet. RPNet was first presented in [47], and regards random patches taken from images as convolution kernels. RPNet does not require training and avoids the over-fitting problem. However, the discrimination ability of random patches is not guaranteed and classification maps generated by RPNet are always noisy [48]. In this regard, instead of RPNet features, RPNet–RF features are introduced for HSI classification. These features can be derived from RPNet feature sets for HSIs. For this, RPNet feature sets are subjected to dimension reduction using PCA and the extracted components are filtered using the RF procedure from [49]. During the HSI classification, the RPNet–RF features are stacked with the spectral HSI features and the obtained spectral-spatial feature vectors are classified by SVM. 

The main contributions of this paper are as follows:

(1) RPNet is combined with RF to generate RPNet–RF features for HSI classification, which have more discrimination power than RPNet and RF features.
(2) A method is proposed, which uses RPNet–RF features for SVM classification of HSIs. The proposed method is not time-consuming, because RPNet does not require any training, and RF can be implemented in real-time.
(3) Using experiments with three widely known datasets and small training samples for each class, it is shown that the proposed RPNet–RF method gives good classification results and outperforms other advanced HSI classification methods (including few-shot learning methods) in terms of overall accuracy and Kappa coefficient.

The remainder of this paper is organized as follows: Section 2 briefly describes the proposed RPNet–RF method. Subsequently, Section 3 exhibits experimental results and their analysis. Finally, some conclusions are drawn in Section 4.

## 2. Method

**Figure 1:**
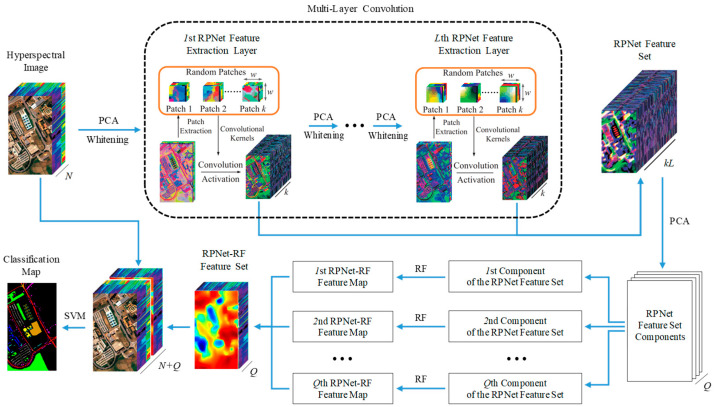
Schematic of the proposed RPNet–RF method for HSI classification.

This Section introduces the RPNet–RF method for HSI classification (Figure 1). The proposed classification method consists of the following steps: (1) RPNet feature extraction, (2) RPNet–RF feature extraction, (3) combining HSI spectral features and RPNet–RF features, and (4) SVM classification by spectral and RPNet–RF features. 

### 2.1. RPNet Feature Extraction 

At the first step of the HSI classification by the proposed method, deep features are extracted from the original HSI *H* using RPNet proposed in [47]. In order to obtain the first layer RPNet features, the following actions are performed:

PCA is first applied to *H,* and only the first *p* PCs are extracted. The dimension-reduced data can be denoted as $H_{P}\in R^{r\times c\times p}$, where r and c are the number of rows and columns of $H_{P}$, respectively. After that, to decrease the correlation between different bands of $H_{P}$ and obtain a similar variance for the bands, the whitening operation is applied to $H_{P}$ [50]. The obtained whitening data can be denoted by $H_{W}\in R^{r\times c\times p}$.

Convolution operations in RPNet are performed using random patches taken from the whitening data $H_{W}$ and regarded as convolution kernels. To obtain *k* random patches, *k* pixels are randomly selected from $H_{W}$, and a patch is taken from around each pixel. Thus, if *k* random patches $P_{1},\;P_{2},\ldots ,P_{k}\;\in R^{w\times w\times p}$ are taken, then *k* feature maps can be generated by convolving the whitening data $H_{W}$ with the random patches:

(1) $$M_{i}=\sum_{j=1}^{p}H_{W}^{\left(j\right)}*P_{i}{}^{\left(j\right)},\;i=1,2,\ldots ,k,$$

where ∗ denotes the 2D convolution operator,$M_{i}\in R^{r\times c}$ is the *i*th feature map, $H_{W}^{\left(j\right)}\in R^{r\times c}$ is the *j*th dimension of $H_{W}$, and $P_{i}{}^{\left(j\right)}\in R^{w\times w}$ is the *j*th dimension of the *i*th random patch.

The obtained feature maps $M_{1},\;M_{2},\;\ldots ,M_{k}$ are stacked into $M\in R^{r\times c\times k}$. After that, *M* is reshaped to 2D matrix $M\in R^{rc\times k}$ in order to further apply the activation function. For improving the sparsity of features from M, the rectified linear units (ReLU) are used as the activation function. Using ReLU, features in the first layer are defined as:

(2) $$F^{\left(1\right)}=\max(0,M-D)$$

where $D\in R^{rc\times k}$ is the mean matrix composed by *k* replications of $d_{2}$, and $d_{2}\in R^{rc\times 1}$ is the mean vector of *M* in the second dimension. Finally, $F^{\left(1\right)}\in R^{rc\times k}$ is reshaped to $F^{\left(1\right)}\in R^{r\times c\times k}$.

The second layer RPNet features are obtained by assuming that $F^{\left(1\right)}$ is a new input *H* and performing the first-layer actions. In a similar manner, features in the *l*th layer can be obtained for all l∈[2,L], where *L* is the network depth. Thus, if $F^{\left(l-1\right)}$(*l* ≥ 2) is the (*l* – 1)th layer features, then *l*th layer features $F^{\left(l\right)}$ can be obtain by assuming that $F^{\left(l-1\right)}$ is a new input *H* and using the feature extraction process from the first layer. 

Finally, the RPNet feature set *F* is formed by combining features from all layers, i.e., $F=\{F^{\left(1\right)},F^{\left(2\right)},\ldots ,F^{\left(L\right)}\}$. 

### 2.2. RPNet–RF Feature Extraction

As follows from Figure 1, the RPNet feature set *F* is used to extract so-called RPNet–RF features. For this, *F* is first subjected to dimension reduction through PCA, and only first *Q* PCs are extracted. The number *Q* of the extracted PCs is defined such that these components preserve 99.95% of the variance of the RPNet feature set.

After that, for each of the extracted PCs, edge-preserving filtering is performed using the transform domain recursive filter [49]. This filter provides that two pixels on the same side of a strong edge have close coordinates, while pixels on opposite sides of a strong edge are far apart. For a discrete 1D signal $I[n]=I(x_{n})$, a recursive edge-preserving filtering can be defined in the transformed domain $\Omega_{\omega }$ as:

(3) $$J[n]=(1-a^{d})I[n]+a^{d}J[n-1]$$

where J[n] is the filtered result, $a=e^{-\sqrt{2}/\delta_{s}}\in [0,1]$ is a feedback coefficient with the spatial parameter δ*_s_*, and $d=ct(x_{n})-ct(x_{n-1})$ is the distance between neighbor samples $x_{n}$ and $x_{n-1}$ in the transform domain $\Omega_{\omega }$, $ct:\Omega \to \Omega_{\omega }$ is a domain transform (DT) used to compute the distance *d*. 

**Figure 2:**
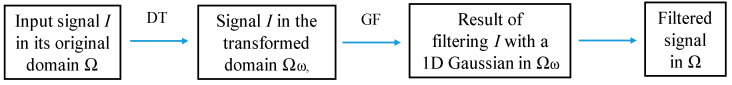
1D edge-preserving filtering using DT *ct*(*u*). GF is the Gaussian filter.

The DT ct(u) for $u\in \Omega_{\omega }$, as follows from [49], can be defined as:

(4) $$ct(u)=\int_{0}^{u}1+\frac{\delta_{s}}{\delta_{r}}|I^{\prime }(x)|dx$$

where *I’*(*x*) is the derivative of *I*(*x*), and δ*_s_* and δ*_r_* are the spatial and range parameters. Figure 2 shows the use of the DT for filtering an 1D signal *I*. 

Since the extracted PCs are 2D images, the 1D filtering operation for each of the PCs is performed separately along each PC dimension iteratively. In other words, 1D filtering is first performed along each PC row, then along each PC column. As shown in [49], three iterations of 1D filtering are sufficient to obtain satisfactory filtering results.

The influence of two parameters δ*_s_* and δ*_r_* on filtering results was analyzed in [49,51]. As δ*_s_* and δ*_r_* increase, the smoothing effect of filtering results becomes more obvious. Moreover, the filtering result will tend to be extremely smooth, if δ*_r_* becomes relatively large (i.e., δ*_r_* = 2). In this case, only a little useful information is preserved. In contrast, when δ*_s_* tends to infinity (e.g., δ*_s_* = 1000), the recursive filter does not produce unlimited smoothing. 

The features obtained using the feature extraction procedure described above are derived from the RPNet feature set using the RF procedure. In this regard, these features can be called as RPNet–RF features.

### 2.3. SVM Classification by Spectral and RPNet–RF Features

At the last step of the HSI classification, the RPNet–RF features obtained for the HSI *H* are stacked with the spectral features of *H*. Since *H* contains *N* spectral band, and feature vectors from the RPNet–RF feature set are *Q-*dimensional, the resulting feature set *Z* consists of (*N* + *Q*)-dimensional feature vectors. 

To classify the resulting high-dimensional vectors of spectral and RPNet–RF features, the SVM classifier is used. SVM aims to explore the optimal separable hyperplane between various classes, and has shown robust performance in solving the high-dimensional and few-shot learning problems [52,53]. Before HSI classification by SVM, all features from *Z* are standardized. Finally, the standardized features are used to predict the class labels through a SVM classifier. To obtain good classification results, Gaussian kernels and fivefold cross-validation can be used for SVM classification. The procedure for HSI classification by the proposed method is summarized in Algorithm 1.
**Algorithm 1** HSI classification via the proposed RPNet–RF method.**Input:** the HSI data *H*; the number of PCs *p*; the network depth *L*; the number of random patches *k*; the size of random patches *w*; the spatial and range standard deviations δ*_s_* and δ*_r_*.**Output:** Predicted class label.**For** layer *l* = 1:*L*
 1:Apply PCA to extract the first *p* PCs of *H*. 2:Calculate the whitening data $H_{W}$. 3:Extract *k* random patches from the whitening data $H_{W}$. 4:Obtain RPNet features $F^{\left(l\right)}$ by (1)–(2) (if l=1) and (3)–(4) (if l>1). 5:If *l* < *L*, renew *H* by $F^{\left(l\right)}$.
**End**
6:Form the feature set *F* by combining $F^{\left(1\right)}$, …, $F^{\left(L\right)}$.7:Apply PCA to extract the first *Q* PCs *C*_1_, …, *C_Q_* of *F*, which explain 99.95% of the variance of *F*.
**For** *q* = 1:*Q*
 8:Perform the transform domain RF for *C_q_*.
**End**
9:Combine the obtained RPNet–RF features with the HSI spectral features to make up the final features.10:Standardize the features.11:Classify the whole image via SVM.


As can be seen from Algorithm 1, the proposed HSI classification method is easy to implement. In addition, the RPNet–RF method has a small number of parameters to be determined. It should also be noted that the RPNet–RF feature extraction procedure is not time-consuming. This is due to the following facts: RPNet does not require any training [47], and RF can be implemented in real-time [49].

## 3. Experiments

### 3.1. Dataset Description

Experimental studies were carried out on the Pavia University (PU) dataset, the Indian Pines (IP) dataset, and the Kennedy Space Center (KSC) dataset.

The PU dataset (Table 1) was acquired by the reflective optics system imaging spectrometer (ROSIS) over an urban area surrounding the University of Pavia (Italy). This dataset has 103 spectral bands of size 610 × 340 pixels. It has a spectral coverage from 0.43 to 0.86 µm, and a spatial resolution of 1.3 m. The ground truth of the PU dataset contains nine classes, most of which are man-made building objects.

The IP dataset (Table 2) was recorded by the airborne visible infrared imaging spectrometer (AVIRIS) over an agricultural area of Northwestern Indiana in 1992. The image has 220 bands with a spatial resolution of 20 m per pixel. After removing the water absorption channels, 200 bands are available. The ground truth of the IP dataset contains 16 land cover classes, and most of these classes are different types of crops.

The KSC dataset (Table 3) was gathered by AVIRIS in 1996, and has 224 bands. The size of the dataset is 512 × 614. The band’s visible and infrared spectra range from 400 nm to 2500 nm, and the ground resolution of this dataset is 18 m. Due to water absorption, some affected and low signal-to-noise bands were abandoned and 176 spectral bands were extracted. The KSC dataset ground truth contains 13 upland and wetland classes.

### 3.2. Experimental Setup and Evaluation Metrics 

All experiments in this paper were performed with an Intel Core i5-11400F 4.2-GHz processor with 32 GB of RAM and a NVIDIA GT730 graphic card. For the HSI classification by the proposed method, a MATLAB R2021b environment was used, and SVM parameters *c* and γ were set to 1024 and 0.01, respectively (our source code is available at https://github.com/UchaevD/RPNet-RF, accessed on 31 October 2022). To evaluate the classification performance in our experimental studies, the following evaluation metrics were used [54]: class accuracy (CA), overall accuracy (OA), average accuracy (AA), and Kappa coefficient. Here, CA is the percentage of correctly classified pixels for each class, which refers to the user’s accuracy; OA is the percentage of correctly classified pixels; AA is the mean of the percentage of correctly classified pixels for each class, the Kappa coefficient is the percentage of correctly classified pixels corrected by the number of agreements that would be expected purely by chance. CA, OA, AA, and Kappa coefficient values were obtained by averaging estimates from several independent experiments.

### 3.3. Effect of Parameter Values on Classification Performance 

As follows from Algorithm 1, results of HSI classification by the proposed method depend on values of the following parameters: *p* is the number of PCs extracted from the HSI, *L* is the network depth (the number of RPNet convolutional layers), *k* is the number of random patches, *w* is the size of patches, and δ*_s_* and δ*_r_* are the spatial and range standard deviations. In this regard, the influences of *p*, *L*, *k, w*, δ*_s,_* and δ*_r_* on the classification performance were analyzed. 

**Figure 3:**
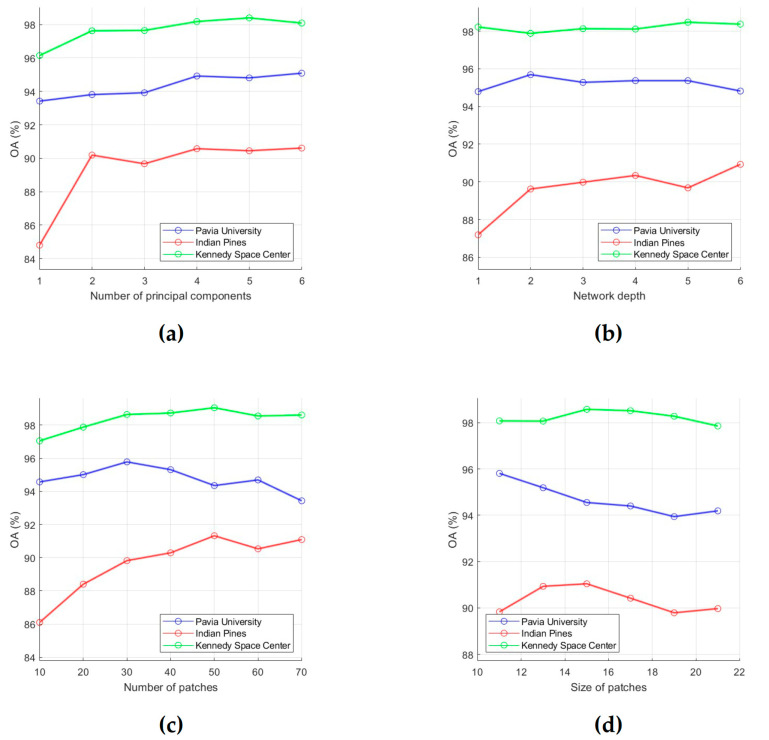
Effect of number of PCs (**a**), network depth (**b**), number of patches (**c**), and size of patches (**d**) on classification OA. OA values were obtained by averaging accuracy estimates obtained after 20 independent runs.

**Figure 4:**
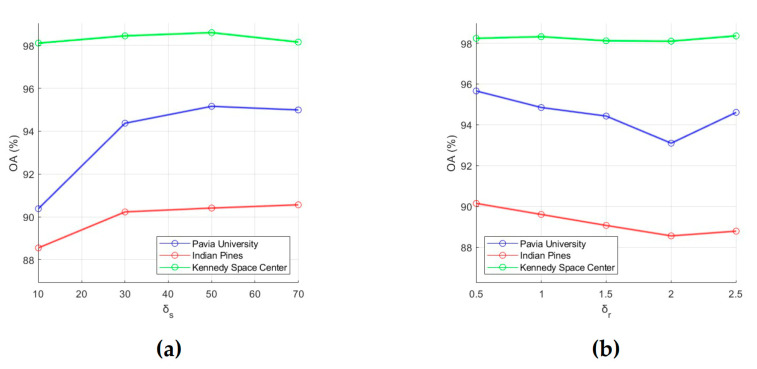
Effect of RF parameters $\delta_{s}$ (**a**) and $\delta_{r}$ (**b**) on classification OA. OA values were obtained by averaging accuracy estimates obtained after 20 independent runs.

To evaluate the classification performance, 15 training samples for each class were randomly selected from the ground truth data, and OA was used as an evaluation metric. First, for each of the six analyzed parameters, intervals of most acceptable values were chosen, which are $I_{p}=[1,6]$, $I_{L}=[1,6]$, $I_{k}=[10,70]$, $I_{w}=[11,21]$, $I_{\delta_{s}}=[10,70]$, and $I_{\delta_{r}}=[0.5,2.5]$. After that, the influence of each parameter on OA values was analyzed. For this, we fixed value of one parameter and changed values of other parameters (values for *p*, *L*, *k*, *w*, δ*_s_* and δ*_r_* were taken from intervals $I_{p}$, $I_{L}$, $I_{k}$, $I_{w}$, $I_{\delta_{s}}$, and $I_{\delta_{r}}$, respectively). As a result, those values for the analyzed parameters were selected, which provide the minimum value of OA (i.e., this is the minimum of a function of six variables). In order to illustrate the effect of parameter values on OA, dependencies of OA values on parameter values are shown in Figure 3 and Figure 4. Each of these plots was obtained under the condition that values of other analyzed parameters are set equal to those found.

As a result of the analysis of effect of parameter values on classification performance, the following conclusions can be drawn:

(a) With an increase in the number of PCs, the classification accuracy first tends to increase, then to a stable change. In particular, as can be seen from Figure 3a, after the number of PCs reaches 4, OA values for the PU, IP, and KSC datasets do not change significantly after that. Thus, we can set the parameter *p* as 4.

(b) The network depth *L* is that parameter, which has various effect on classification accuracy. The reason of this phenomenon is the following: as the layer gets deeper, the network not only extracts more abstract and robust features, but also leads to information loss. As can be seen from Figure 3b, when *L* increases from 1 to 6, OA changes only slightly for the PU and KSC datasets. In contrast, for the IP dataset, OA grows as long as L≤4. Therefore, we can set *L* equal to 4.

(c) Too few random patches cannot provide a high classification accuracy. Figure 3c shows that *k* can be taken equal to 50, since at *k* = 50 OA reaches its highest values for the IP and KSC datasets (the OA values for the PU dataset fluctuate around 95% when *k* increases from 10 to 60).

(d) A larger *w* also increase the possibility of over-smoothing phenomenon. Dependencies of OA on *w* values, which are presented in Figure 3d, show that OA for the PU dataset decreases over the interval [11,19], while OA for the IP and KSC datasets reaches a maximum value at *w* = 15. Thus, we can conclude that 15 is better suited for *w*.

(e) Small values of δ*_s_* provides low classification accuracy because it means that only very limited local spatial information is considered in the feature extraction process. Plots in Figure 4a are confirmed this conclusion and show that OA grows as δ*_s_* values increases from 10 to 50, then (for δ*_s_* > 50) decreases slightly for the PU and KSC datasets or slightly increases for the IP dataset. Based on this fact, we can set δ*_s_* to 50.

(f) A large δ*_r_* will reduce classification accuracy because recursive filtering with a large δ*_r_* will produce extremely smooth feature images [51]. In this regard, we propose to take 0.5 for δ*_r_*. As can be seen from Figure 4b, for the PU, IP, and KSC datasets, OA values decrease as δ*_r_* increases from 0.5 to 2.

Based on the parameter analysis results, we have summarized the recommended parameter values in Table 4.

### 3.4. Classification Results

Classification results by our method were obtained for the PU, IP, and KSC datasets. For this, 15 labeled training samples for each class were taken from the ground truth data, and parameter values from Table 4 were used for classification. 

To demonstrate advantages of the proposed RPNet–RF method, the obtained HSI classification results were compared with results obtained by other recently proposed HSI classification methods that are adopted to solve the few-short learning problem. For the comparison with the proposed method, the following HSI classification methods were chosen: IFRF [51], 3D-CNN [16], RPNet [47], CA-GAN [30], DCFSL [37], 3D VS-CNN [35], S-DMM [39], and TC-GAN [42]. All of these methods (excluding IFRF) are deep-learning methods and produce good classification results for small labeled training samples. A detailed overview of some features and parameter setting for the compared methods is presented below. 

For IFRF [51], it is necessary to determine the spatial standard deviation (δ*_s_*), the range standard deviation (δ*_r_*), and the number of IFRF features (*k*). In our experiments, for δ*_s_*, δ*_r_*, and *k*, we set the values recommended in [51]; namely, δ*_s_* and δ*_r_*, which characterize results of filtering, were set to 200 and 0.3, respectively, and *k* was set to 20, since this provides good classification accuracy and relatively low computing burden. 

In 3D-CNN [16], two 3D convolution layers (C1, C2), one fully connected layer and one classification layer are used. For 3D convolution layers, 3D kernels should be determined (so, in our experimental studies on the PU and IP datasets, C1 contained two 3 × 3 × 7 kernels, and C2 contained four 3 × 3 × 3 kernels). By applying 3D kernels to HSIs, 3D-CNN can learn the local signal changes in both the spatial and spectral dimension of the feature cubes, exploiting important discrimination information for classification.

For RPNet [47], the following parameters should be manually set: the number of PCs extracted from the HSI (*p*), the network depth (*L*), the number of random patches (*k*), and the size of patches (*w*). In our experimental studies, to obtain classification maps comparable to RPNet–RF maps, the above-mentioned RPNet parameters were assigned the values used for the proposed RPNet–RF method, i.e., *p =* 4, *L =* 4, *k =* 50, and *w =* 15. 

In CA-GAN [30], four transposed convolutional layers (TCL) are used for the generator. Each TCL is constructed based on the convolutional 5 × 5 kernel. Before each TCL, the joint spatial-spectral hard attention module is incorporated into the generator. The sizes of generated feature maps inputting to each attention module are 2 × 2 × 128, 4 × 4 × 64, 7 × 7 × 32, and 14 × 14 × 16, respectively. In the discriminator of CA-GAN, real samples and generated samples of the same size of 27 × 27 × 20 are input. To extract hierarchical features, four convolutional layers with the convolutional kernel size of 5 × 5 are used in the discriminator. The sizes of the extracted feature maps are 14 × 14 × 16, 7 × 7 × 32, 4 × 4 × 64, and 2 × 2 × 128, respectively. 

For DCFSL [37], 9 × 9 neighborhoods are chosen as the spatial size of the input cubes. DCFSL is trained via Adam optimizer, and the number of the training iterations can be set to 10 000, which is enough to train the network. Since labeled training data of each class is used in DCFSL to learn a C-class classification model, C was set to the class number of the target dataset (i.e., C = 9 for the PU dataset, C = 16 for the IP dataset, and C = 13 for the KSC dataset). 

In 3D VS-CNN [35], the SVM is used as a selector to iteratively select some of the most valuable training samples. To construct valuable training samples, active learning is used. After that, spectral-spatial HSI information is extracted by 3D CNN.

For S-DMM, as follows from [39], the following two parameters should be manually set: patch size of a pixel sample (*P*), and number of convolution filters in the network (*D*). In our experiments on the test datasets, *P* was fixed to 5, and *D* was set to 64.

In TC-GAN [42], dual-channel blocks are used to extract spatial and spectral features. Each of the blocks will learn the spatial information and spectral information around the label pixel, and compress the input 3D cube with size of *W* × *W* × *N* (here *W* is used to set the width and height of the cube, and *N* is the number of spectral bands) to an appropriate size. In particular, for the IP dataset, as shown in [42], the 3D cube can have a size of 15 × 15 × 200. 

IFRF and RPNet classification results were obtained by MATLAB toolboxes from the sources: https://www.mathworks.com/matlabcentral/fileexchange/68966-a-demo-for-hyperspectral-image-classification?s_tid=prof_contriblnk (accessed on 15 October 2022), https://github.com/YonghaoXu/RPNet (accessed on 17 October 2022). Other HSI classification results were taken from [42]. 

**Figure 5:**
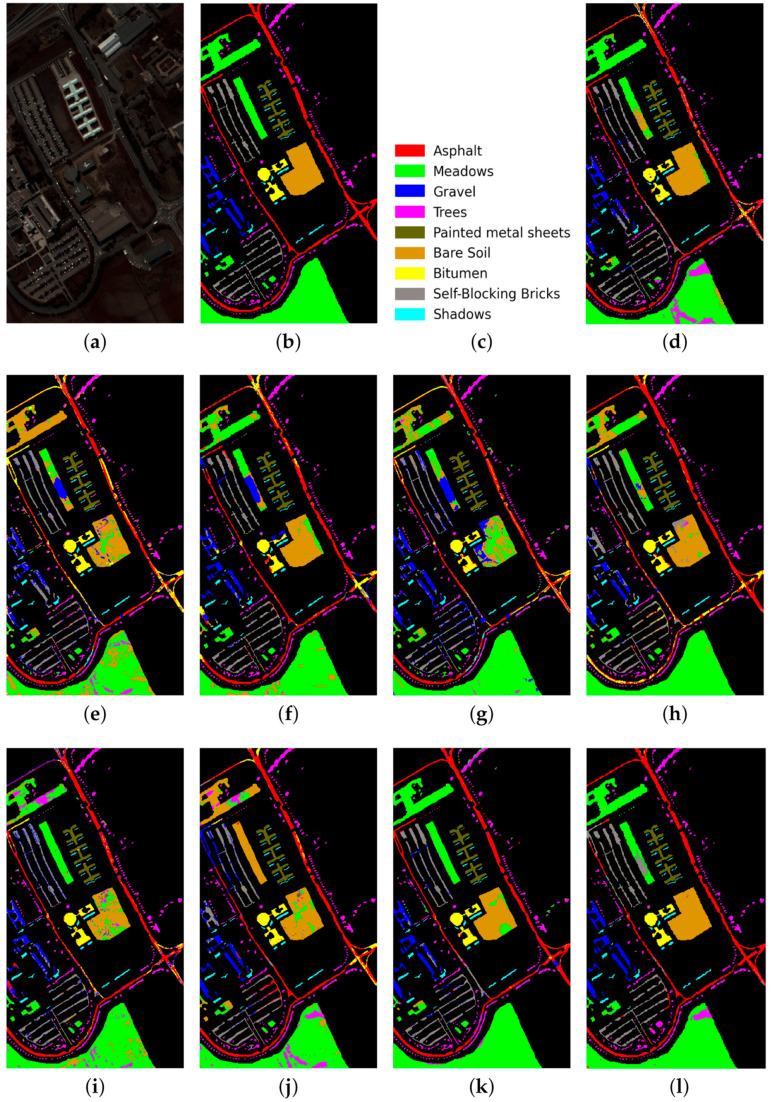
PU dataset: (**a**) false-color image, (**b**) ground truth map, (**c**) legend. Classification maps obtained by the compared methods on the PU dataset: (**d**) IFRF, (**e**) 3D-CNN, (**f**) RPNet, (**g**) CA-GAN, (**h**) DCFSL, (**i**) 3D VS-CNN, (**j**) S-DMM, (**k**) TC-GAN, and (**l**) RPNet–RF. Fifteen labeled samples per class were randomly selected for training.

**Figure 6:**
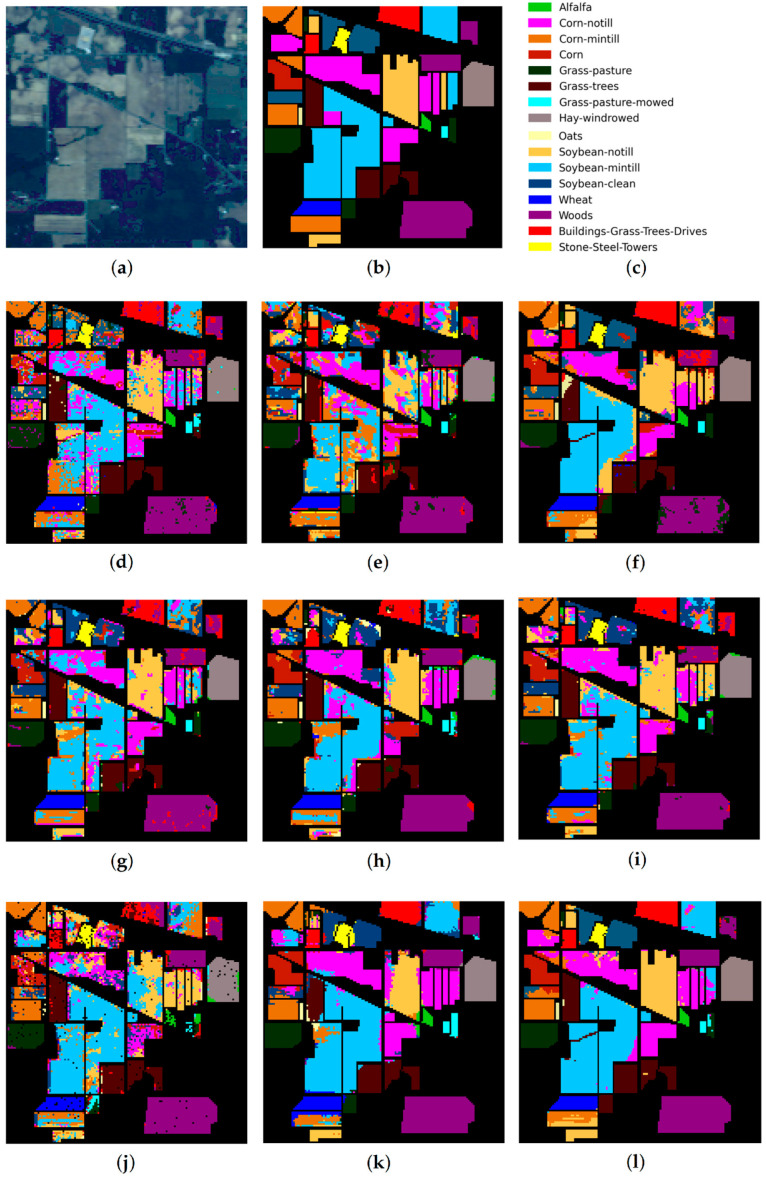
IP dataset: (**a**) false-color image, (**b**) ground truth map, (**c**) legend. Classification maps obtained by the compared methods on the IP dataset: (**d**) IFRF, (**e**) 3D-CNN, (**f**) RPNet, (**g**) CA-GAN, (**h**) DCFSL, (**i**) 3D VS-CNN, (**j**) S-DMM, (**k**) TC-GAN, and (**l**) RPNet–RF. Fifteen labeled samples per class were randomly selected for training.

**Figure 7:**
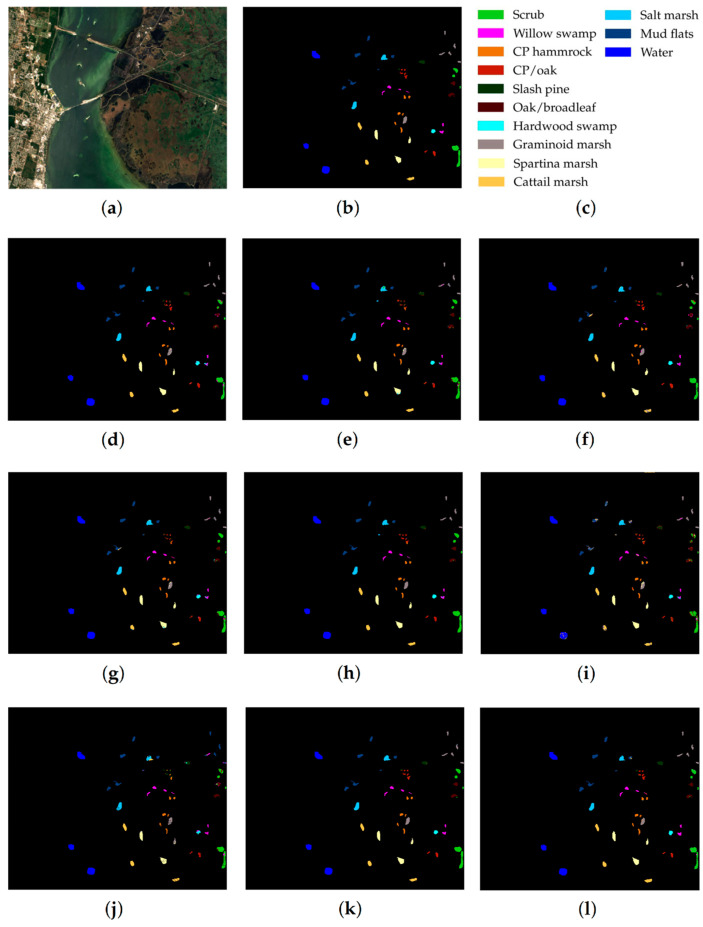
KSC dataset: (**a**) false-color image, (**b**) ground truth map, (**c**) legend. Classification maps obtained by the compared methods on the KSC dataset: (**d**) IFRF, (**e**) 3D-CNN, (**f**) RPNet, (**g**) CA-GAN, (**h**) DCFSL, (**i**) 3D VS-CNN, (**j**) S-DMM, (**k**) TC-GAN, and (**l**) RPNet–RF. Fifteen labeled samples per class were randomly selected for training.

For each of the compared methods, classification results were obtained 10 times, then means for CA, OA, AA, and Kappa were estimated. The resulting accuracy estimates for the test datasets are shown in Table 5, Table 6 and Table 7. Figure 5, Figure 6 and Figure 7 preceding these tables show classification maps obtained by all compared methods for the PU (Figure 5d–l), IP (Figure 6d–l), and KSC (Figure 7d–l) datasets. 

**Figure 8:**
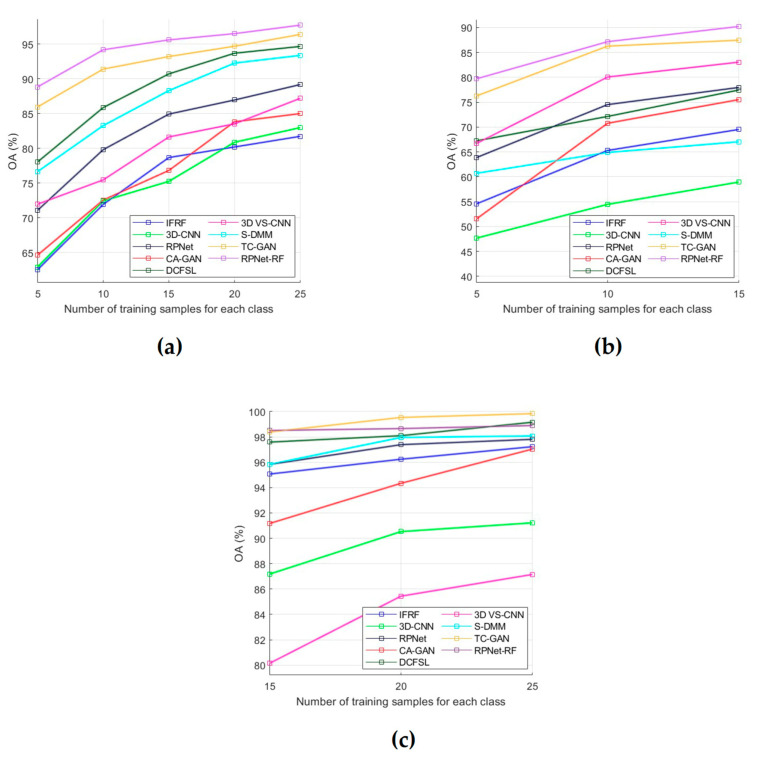
OA for each of the compared methods as a function of the number of labeled training samples per class: (**a**) PU dataset, (**b**) IP dataset, and (**c**) KSC dataset.

During the performed experimental studies, the influence of the number of training samples on the classification performance was also analyzed. For this, OA was used as an evaluation metric, and dependencies of OA means on the number of training samples were plotted for each of the test datasets (Figure 8). The OA means were obtained by OA values for classification results in 10 independent experiments. A detailed analysis of all obtained results is presented in the next section.

## 4. Discussion

Based on the analysis of classification results obtained for the PU, IP, and KSC datasets (Table 5, Table 6 and Table 7 and Figure 5, Figure 6, Figure 7 and Figure 8), the following conclusions can be drawn.

As follows from Table 5, Table 6 and Table 7, the proposed RPNet–RF method outperforms other HSI classification methods in terms of OA and Kappa. In particular, Table 5 with the HSI classification results obtained for the PU dataset shows that our method, which combines RPNet with RF, outperforms the RPNet method (by 11% in OA) and the IFRF method (by 7% in OA), respectively. Moreover, it can be noted that our method produces better classification results in cases where the ratio between the number of test and training samples is large. In particular, for the PU dataset classes Asphalt, Meadows, and Bare Soil, where the ratio between the number of test and training samples is huge, our method gives the best classification accuracies (97.37%, 99.37%, and 99.92%, respectively). In general, it can be said that when 15 training samples for each class is randomly selected from the ground truth, then the proposed RPNet–RF method achieves the highest classification accuracy for four PU dataset classes, four IP dataset classes, and three KSC dataset classes. A significantly lower RPNet–RF classification accuracy were obtained for the PU dataset class Trees and IP dataset classes Grass-pasture-mowed and Oats. This can be explained by the following facts. In some cases, RPNet–RF cannot distinguish trees from meadows. A similar conclusion can also be drawn for such HSI classification methods as IFRF and 3D VS-CNN (see Figure 5i-j). Grass-pasture-mowed and Oats, as can be seen from Table 2, are the smallest IP dataset classes for which the ratio between the number of test and training samples is very small. As a result, user’s accuracy for these classes is greatly reduced when a few pixels from other classes are included in the classes. As can be seen from Figure 5l, this is true for the RPNet–RF classification map, where the Grass-pasture-mowed class contains some samples from the Grass-pasture class, and class Oats contains a few samples from Grass-trees. 

Figure 5, Figure 6 and Figure 7 with classification maps obtained by compared methods show that the proposed method is much better in removing “noisy pixels” than such HSI classification methods as 3D-CNN, CA-GAN, DCFSL, 3D VS-CNN, and S-DMM. TC-GAN that combines a generative adversarial network, transformer encoder, and convolution block also produces classification maps without noise. However, it cannot distinguish well between certain classes (e.g., PU dataset classes Meadows and Bare Soil, and IP dataset classes Oats and Soybean-clean).

Some conclusions about the RPNet–RF performance can be drawn from the analysis of the influence of training sample number on the classification accuracy (Figure 8). In particular, as follows from Figure 8a,b, for the PU and IP datasets, our method outperforms other HSI classification methods that were used for comparison. For the KSC dataset (Figure 8c), when the number of training samples for each class is greater than 15, our method provides slightly lower OA values than TC-GAN and DCFSL. However, if 15 training samples per class are used, the proposed method outperforms TC-GAN and DCFSL. Therefore, we can say the following: when the number of training samples selected for each classes is quite limited, then RPNet–RF is more suitable for HSI classification than other methods from Figure 8.

## 5. Conclusions

In this paper, we propose a new HSI classification method that combines RPNet and RF in a unified framework. This method consists of the following steps: RPNet feature extraction, RPNet–RF feature extraction, combining HSI spectral features and RPNet–RF features, and SVM classification by spectral and RPNet–RF features. The proposed RPNet–RF classification method is easy to implement, has a small number of parameters to be determined (number of PCs, network depth, number of random patches, size of random patches, and spatial and range standard deviations), and is not time-consuming (because RPNet does not require any training, and RF can be implemented in real-time).

Experiments with three widely known datasets demonstrate that when the number of training samples per class is limited (from 5 to 25), our method outperforms (in terms of OA and Kappa) other advanced HSI classification methods, including recently proposed few-shot learning methods. Moreover, it is established that the RPNet–RF classification method produces better classification results for such cases when the ratio between the number of test and training samples is large.

In the future, we plan to conduct a comprehensive study to improve the proposed HSI classification method. In particular, we would like to explore the possibilities of using semi-supervised classifiers instead of SVM, which are better suited to solve the few-shot learning problem.

## Figures and Tables

**Table 1:** Ground truth classes and per-class samples for the PU dataset.

Class No.	Class Name	Labeled Samples
1	Asphalt	6631
2	Meadows	18,649
3	Gravel	2099
4	Trees	3064
5	Painted metal sheets	1345
6	Bare Soil	5029
7	Bitumen	1330
8	Self-Blocking Bricks	3682
9	Shadows	947

**Table 2:** Ground truth classes and per-class samples for the IP dataset.

Class No.	Class Name	Labeled Samples
1	Alfalfa	46
2	Corn-notill	1428
3	Corn-mintill	830
4	Corn	237
5	Grass-pasture	483
6	Grass-trees	730
7	Grass-pasture-mowed	28
8	Hay-windrowed	478
9	Oats	20
10	Soybean-notill	972
11	Soybean-mintill	2455
12	Soybean-clean	593
13	Wheat	205
14	Woods	1265
15	Buildings-Grass-Trees-Drives	386
16	Stone-Steel-Towers	93

**Table 3:** Ground truth classes and per-class samples for the KSC dataset.

Class No.	Class Name	Labeled Samples
1	Scrub	761
2	Willow swamp	243
3	CP hammock	256
4	CP/oak	252
5	Slash pine	161
6	Oak/broadleaf	229
7	Hardwood swamp	105
8	Graminoid marsh	431
9	Spartina marsh	520
10	Cattail marsh	404
11	Salt marsh	419
12	Mud flats	503
13	Water	927

**Table 4:** Recommended values of parameters for the proposed classification method.

Parameter	Description	Value
*p*	Number of PCs	4
*L*	Network depth	4
*k*	Number of patches	50
*w*	Size of patches	15
$\delta_{s}$	Spatial standard deviation	50
$\delta_{r}$	Range standard deviation	0.5

**Table 5:** Classification accuracies for the proposed and compared HSI classification methods on the PU dataset (the best accuracy in each row is shown in bold). Fifteen labeled samples per class were randomly selected for training.

Class No.	IFRF	3D-CNN	RPNet	CA-GAN	DCFSL	3D VS-CNN	S-DMM	TC-GAN	RPNet– RF
1	95.42	70.41	91.70	60.16	74.55	83.27	96.97	89.07	**97.37**
2	97.78	73.10	95.44	72.83	97.20	76.96	81.15	97.57	**99.37**
3	74.73	73.80	62.90	98.03	80.57	81.91	92.69	67.08	**98.19 **
4	86.70	89.37	97.05	89.44	94.62	86.86	**97.50**	88.03	79.86
5	99.08	96.39	99.74	99.70	**100.0**	99.55	**100.0**	**100.0**	98.85
6	75.34	69.68	64.15	79.94	90.37	82.81	84.73	93.80	**99.92**
7	64.12	86.46	58.31	90.04	92.47	77.94	97.71	**99.47**	94.82
8	81.23	77.09	80.30	81.95	81.62	**93.58**	93.23	93.07	86.67
9	99.52	86.05	99.72	97.32	**100.0**	71.84	99.89	96.67	99.58
OA (%)	88.38	75.24	84.92	76.81	90.71	81.63	88.30	93.20	**95.60**
AA (%)	85.99	80.26	83.26	76.94	90.20	83.86	93.76	91.60	**94.96**
Kappa (%)	84.97	68.43	80.52	71.02	87.73	76.46	84.90	91.00	**94.27**

**Table 6:** Classification accuracies for the proposed and compared HSI classification methods on the IP dataset (the best accuracy in each row is shown in bold). Fifteen labeled samples per class were randomly selected for training.

Class No.	IFRF	3D-CNN	RPNet	CA-GAN	DCFSL	3D VS-CNN	S-DMM	TC-GAN	RPNet– RF
1	77.71	83.87	94.16	**100.0**	**100.0**	90.32	91.67	**100.0**	93.48
2	62.91	38.08	72.19	61.78	60.79	75.94	47.18	78.77	**81.30**
3	50.08	41.84	57.05	68.22	78.77	85.03	44.88	**92.15**	85.66
4	39.36	52.70	54.84	92.34	94.59	95.95	33.04	**99.10**	83.31
5	73.57	74.79	73.30	82.69	85.68	91.03	78.44	**95.30**	94.14
6	93.29	87.27	92.58	89.51	96.64	**97.34**	92.50	95.94	95.15
7	42.82	**100.0**	53.39	**100.0**	**100.0**	**100.0**	**100.0**	**100.0**	43.98
8	97.73	94.38	99.83	99.78	92.22	97.84	85.26	**100.0**	97.76
9	15.46	**100.0**	39.15	**100.0**	**100.0**	**100.0**	**100.0**	**100.0**	63.83
10	57.32	64.26	67.94	76.28	71.89	80.88	66.74	**86.00**	83.07
11	74.93	41.43	91.75	64.22	65.66	73.32	70.39	81.39	**94.44**
12	50.50	41.70	65.53	78.72	73.18	**88.41**	40.82	73.18	82.96
13	87.94	99.47	93.40	99.47	**100.0**	98.95	99.49	**100.0**	99.10
14	94.64	84.24	96.64	82.32	93.28	84.24	81.35	97.28	**99.77**
15	76.78	70.89	78.08	92.99	87.87	86.52	68.35	83.83	**98.45**
16	86.35	97.44	95.71	92.31	**100.0**	98.72	98.80	**100.0**	97.60
OA (%)	69.52	58.94	77.97	75.52	77.45	83.06	67.04	87.47	**90.23**
AA (%)	67.59	73.27	76.60	81.21	87.54	90.28	74.93	**92.68**	87.12
Kappa (%)	65.70	54.06	75.19	72.69	74.65	80.89	62.44	85.78	**88.87**

**Table 7:** Classification accuracies for the proposed and compared HSI classification methods on the KSC dataset (the best accuracy in each row is shown in bold). Fifteen labeled samples per class were randomly selected for training.

Class No.	IFRF	3D-CNN	RPNet	CA-GAN	DCFSL	3D VS-CNN	S-DMM	TC-GAN	RPNet– RF
1	98.16	89.41	99.12	88.20	96.92	97.15	96.01	**99.87**	99.46
2	93.54	86.40	83.79	85.53	86.40	91.28	88.84	**100.0**	96.20
3	94.38	85.06	94.94	95.02	98.76	80.09	**99.19**	96.68	97.54
4	86.12	54.01	88.47	90.72	82.28	42.29	54.96	86.08	**98.32**
5	79.89	83.56	84.79	90.41	91.78	58.09	80.79	93.84	**95.90**
6	83.66	76.64	92.38	94.39	97.66	70.59	96.35	**100.0**	91.53
7	85.99	**100.0**	98.16	**100.0**	**100.0**	70.00	**100.0**	97.78	**100.0**
8	93.97	92.55	94.28	86.78	**100.0**	62.81	99.29	96.63	98.14
9	96.72	60.59	97.28	86.73	**100.0**	74.55	**100.0**	99.60	98.52
10	94.11	93.32	94.28	86.12	99.74	61.48	**100.0**	99.49	99.06
11	99.75	93.07	**100.0**	92.57	**100.0**	78.68	**100.0**	**100.0**	99.80
12	98.49	93.85	**99.73**	88.52	99.18	78.24	98.99	97.95	99.16
13	99.51	**100.0**	99.85	**100.0**	**100.0**	99.89	**100.0**	**100.0**	99.95
OA (%)	95.07	87.18	95.83	91.17	97.59	80.15	95.83	98.39	**98.51**
AA (%)	92.64	85.27	94.39	84.64	96.36	74.24	93.42	97.53	**97.97**
Kappa (%)	94.51	85.73	95.36	90.20	97.31	77.81	95.35	98.20	**98.33**

## Data Availability

Publicly available datasets were analyzed in this study, which can be found here: http://www.ehu.eus/ccwintco/index.php/Hyperspectral_Remote_Sensing_Scenes (accessed on 10 September 2022).
